# Supplementary material for: Rapid turnover of life-cycle-related genes in the brown algae
Source: Genome Biol. 2019 Feb 14;20:35. doi: 10.1186/s13059-019-1630-6 (PMC6374913; doi:10.1186/s13059-019-1630-6)
Supplement: Supplementary file 3 — Protein alignments, corresponding Gblocks html file and CDS sequences for the lineage conserved gametophyte-biased genes. (ZIP 2109 kb) [file 13059_2019_1630_MOESM3_ESM.zip › Con_GA_alignments/Ec-18_002680.aa_ali.fasta-gb.htm]

Ec-18\_002680.aa.fasta.trans.aa\_ali.fasta


## Gblocks 0.91b Results

Processed file: **Ec-18\_002680.aa.fasta.trans.aa\_ali.fasta**  
Number of sequences: **2**  
Alignment assumed to be: **Protein**  
New number of positions: **217** (selected positions are underlined in blue)

```
                         10        20        30        40        50        60
                 =========+=========+=========+=========+=========+=========+
Ec-18_002680     MAEQEVVSAPDDASRENVETADSAGSGRLQDQPGAADEPEHDVDERK----APPSTRES-
MSTRG.39194.1    MAEEDVVSESYTAC----EDAESVGSGRDEDRSGAADKSAQDEGIDKGADDAPPFTQKSS
                 #############     #############################             


                         70        80        90       100       110       120
                 =========+=========+=========+=========+=========+=========+
Ec-18_002680     ----------ELGGPTSSRGDEGGAPPS-SAGASTRRRDKKKKGRGGGACGGEPAMSVED
MSTRG.39194.1    VAETGASPDDALAGVGATPAPSGAAPVSRGSGTASSKKERKKKGRGG--AEGKPAMSIED
                            #################   ################    #########


                        130       140       150       160       170       180
                 =========+=========+=========+=========+=========+=========+
Ec-18_002680     LFVTISTVQVSGINPPPARVVLTPRSAESCLKHGVNPEILRVRDLDSFWEPGVEPEVQRM
MSTRG.39194.1    LFVTISTVQVSGINPPPARVVLTPRSAEACLKHGVNPEILRVRDLDSFWEPGVEPEVQRM
                 ############################################################


                        190       200       210       220       230       240
                 =========+=========+=========+=========+=========+=========+
Ec-18_002680     RHEAYSQRRHEMMRLCRLERKRLINAEQKQLALSHMAGGAVLTPAQLLDQQKKANATMIE
MSTRG.39194.1    RHEAYSQRRHEMMRLCRQERKRLINAEQKQLALSHMAGGAVLTPAQLLDQQKKANATMIE
                 ############################################################


                        250       260       270       280       290       300
                 =========+=========+=========+=========+=========+=========+
Ec-18_002680     LEERRLEKMQRRQEKELEQLLQWESKQKRVQEENHARAENERRKQEQRLRERNRRQREIA
MSTRG.39194.1    LEERRLEKMQRRQV----------------------------------------------
                 #############                                               


                        310       320       330       340       350       360
                 =========+=========+=========+=========+=========+=========+
Ec-18_002680     EERRLRELKKKALEDAEEVRRQALAKAAHQREQELADERERREKLAKADARNREEERVRK
MSTRG.39194.1    ------------------------------------------------------------
                                                                             


                        370       380       390       400       410       420
                 =========+=========+=========+=========+=========+=========+
Ec-18_002680     AEEHRLQTQRILEDQQFKMQKRMEEMKEQEAERQRKVEEQQAKEKADLRERRSRVKMRIQ
MSTRG.39194.1    ------------------------------------------------------------
                                                                             


                        430       440       450       460       470       480
                 =========+=========+=========+=========+=========+=========+
Ec-18_002680     ENLKMAAMAEEQRKANFFQKHGEHEEKRRIARMRSEEERQLLARQRYLQEKKRMIILDES
MSTRG.39194.1    ------------------------------------------------------------
                                                                             


                        490       500       510       520       530       540
                 =========+=========+=========+=========+=========+=========+
Ec-18_002680     RAEEQARKEAGLARIEQEEEYLHQLLERRARERYIQKERNELQKQLKMENVNRIQRIQEY
MSTRG.39194.1    ------------------------------------------------------------
                                                                             


                        550       560       570       580       590       600
                 =========+=========+=========+=========+=========+=========+
Ec-18_002680     KRLETLRQIKEADRRTEEMATRKEEMINQRRRAALEAKMQKEEITRIMENTKRNQKKAAK
MSTRG.39194.1    ------------------------------------------------------------
                                                                             


                        610       620       630       640       650       660
                 =========+=========+=========+=========+=========+=========+
Ec-18_002680     LIQKAMKRGKSRSDAPRRRRRPAGNNNNNNNNNDSNNWSDENFEGGNDGRSSSANLPATA
MSTRG.39194.1    ------------------------------------------------------------
                                                                             


                        670       680       690       700       710       720
                 =========+=========+=========+=========+=========+=========+
Ec-18_002680     GGQSSGNDRIKSARAATRARGEVDPANKVDLGPPPDAPTLEARLRAAAPSDPSPQPYMSP
MSTRG.39194.1    ------------------------------------------------------------
                                                                             


                        730
                 =========+=====
Ec-18_002680     YENTATPQPSATVTF
MSTRG.39194.1    ---------------
```

```
Parameters used
Minimum Number Of Sequences For A Conserved Position: 2
Minimum Number Of Sequences For A Flanking Position: 2
Maximum Number Of Contiguous Nonconserved Positions: 8
Minimum Length Of A Block: 10
Allowed Gap Positions: None
Use Similarity Matrices: Yes
```

```
Flank positions of the 5 selected block(s)
Flanks: [1  13]  [19  47]  [72  88]  [92  107]  [112  253]  

New number of positions in Ec-18_002680.aa.fasta.trans.aa_ali.fasta-gb:  217  (29% of the original 735 positions)
```
